# Cell line tropism and replication of XMRV

**DOI:** 10.1186/1742-4690-8-S1-A225

**Published:** 2011-06-06

**Authors:** Krishnakumar Devadas, Mohan K H G Setty, Ragupathy Viswanath, Durga S Gaddam, Owen Wood, Shixing Tang, Jiangqin Zhao, Xue Wang, Veeraswamy Ravichandran, Sherwin Lee, Indira K Hewlett

**Affiliations:** 1 Center for Biologics Evaluation and Research, Food and Drug Administration, Bethesda, MD, 20892, USA

## Background

XMRV is a gammaretrovirus closely related to xenotropic murine leukemia viruses (MuLVs). XMRV was first identified in familial cases of prostate cancer tissue using a virus gene array. Although, initial reports have identified XMRV predominantly in the prostate, recent reports of detection of XMRV in blood cells of patients with Chronic Fatigue Syndrome suggests that blood cells could act as a primary target and reservoir for XMRV to help disseminate infection throughout the body. The aim of this study is to elucidate possible routes of transmission and to determine the host range and cellular tropism of XMRV.

## Methods

Culture supernatants containing infectious virus from 22RV-1 or DU145-clone-7 cells were used to infect human cell lines Jurkat, H9, HL60, U937, primary PBMC and monocyte-derived macrophages representing the hematopoietic system. In addition, a variety of epithelial cells, lung epithelial cell line A549 and cervical epithelial cell lines CaSki, HeLa and SiHa were evaluated for infectivity with prostate cancer lines DU145 and LNCaP serving as positive controls. Infected cells were monitored for XMRV replication over a period of 5-7 days. XMRV replication was quantitated by RT-PCR, DNA PCR and real-time PCR. The ability of these cell lines to produce infectious viral particles was also determined.

## Results

Replication of XMRV could be observed in cervical and lung epithelial cells, T-cell lines Jurkat and H9, B-cell line HL60, U937 cells and in primary PBMC and monocyte-derived macrophages. The levels of XMRV transcripts were lower in primary monocytes compared to T-cell lines suggesting less efficient replication in these cells.

## Conclusion

Viral replication could be identified in primary hematopoietic cells and a variety of epithelial cells in addition to the previously described prostate cancer derived cell lines investigated. Viral replication was considerably lower in primary monocytes, suggesting less efficient replication in these cells. These observations will help to further our understanding of XMRV pathogenesis and provide insights into the modes of transmission involved in XMRV infection.

